# Paradigm shift in treatment of coarctation of the aorta?

**DOI:** 10.1007/s12471-020-01536-5

**Published:** 2021-01-11

**Authors:** V. Sojak, M. Hazekamp

**Affiliations:** 1 Heart Lung Center, Leiden University Medical Center, Leiden, The Netherlands; 2 Department of Cardiothoracic Surgery, Leiden University Medical Center, Leiden, The Netherlands

Individuals with coarctation of the aorta (CoA) have had historically poor long-term outcomes with a mean life expectancy of 35 years [1]. Advances in diagnostics, surgical techniques, intensive care and cardiology in the past decades dramatically changed the dismal outlook of these patients. Although reinterventions due to late complications and cardiovascular death are not infrequent, the overall population of patients with CoA shows very good survival up to the age of 50 years [1].

Dijkema et al. present their 20-year experience with treatment of native CoA in 206 children who have been followed for at least 7 years [2]. To assess time-related impact on outcomes, the patients were subdivided in groups corresponding to the following 3 treatment eras: 1987–1995, 1996–2002, and 2003–2010. Surgical procedures were employed in 177 patients. The types of procedures have evolved over time; while the use of CoA resection with end-to-end anastomosis or extended end-to-end anastomosis has remained relatively constant, that of patch angioplasty has substantially decreased, whereas the use of aortic arch repair with cardiopulmonary bypass (CPB) and deep hypothermic circulatory arrest (DHCA) markedly increased over the study period. Furthermore, 29 patients were treated primarily with catheter-based techniques, i.e. balloon dilatation and/or stents (6 patients).

Reinterventions were needed in 83 patients because of re-coarctation (94%) and aortic aneurysm (6%). A significantly higher (doubled) risk of reintervention was noted in patients undergoing catheter-based treatment, and in those undergoing surgery before 3 months of age. The differences in reintervention rates did not differ among particular treatment eras.

Hypertension was noted in 20% of patients and occurred similarly across all three treatment periods. Nevertheless, its rate in non-surgical patients was significantly higher (at least doubled) than that in patients operated on.

Aneurysms were found in 8% of patients, both after surgical and catheter-based treatment, and no differences were observed among particular treatment eras.

The reported outcomes are in accordance with previously published data. In a multi-centre US study analysing survival (median follow-up of 17.7 years) of 2424 patients undergoing coarctation repair in the period 1982–2003, Oster et al. reported an excellent long-term survival (97.5% at 1 year and 95.6% at 20 years) and a small ongoing risk of mortality into early adulthood. Patients at higher risk were those with body weight under 2.5 kg, genetic defects, or those operated on before 1990. The type of surgery was not associated with significant differences in survival [3]. Another large retrospective study of 834 patients undergoing CoA repair with median follow-up of 27 years showed overall survival of 99%, 88% and 65% at 30, 50 and 70 years respectively. The reintervention rate was 40%, and young age and catheter-based treatment were identified as risk factors. Up to 60% of patients have developed hypertension over the time irrespective of status of repair. These data suggest that despite the optimistic outlook for coarctation repair survivors during the first 30 years, potential late risks such as re-coarctation, aneurysm, hypertension and related cardiovascular and cerebrovascular disease significantly reduce their life expectancy compared with matched population and call for life-long surveillance [4].

Dijkema et al. imply a recent shift in treatment strategy favouring aortic arch repair with the use of CPB and DHCA in infants due to reported inconsistent aortic arch growth after isolated coarctation repair, while in older children with simple CoA they advocate balloon dilatation and/or stenting. Nevertheless, this strategy has not translated into more favourable freedom from reintervention, aneurysm formation, or hypertension. Bearing in mind potential neurodevelopmental consequences and complications of CPB and DHCA in newborns and small infants, this leads us to raise the issue of the selection of appropriate surgical techniques in patients with CoA. We believe that coarctation resection and end-to-end, or extended end-to-end, technique is usually adequate in patients with simple CoA. Only rarely a reverse subclavian flap is needed to additionally enlarge the longer and hypoplastic distal transverse arch. In accordance, a large study of long-term outcomes of coarctation repair suggests that extended end-to-end anastomosis is the optimal surgical approach for infants and young children with simple CoA. Even with associated transverse arch hypoplasia, the repair through left thoracotomy has a low mortality, low reintervention rate and low incidence of hypertension. Median sternotomy should be considered for patients with significant ventricular septal defect (VSD) or other cardiac anomalies and proximal or distal transverse arch hypoplasia [5]. We also believe that patients with complex CoA (in association with VSD and/or aortic arch hypoplasia) require aortic arch and VSD repair using CPB and DHCA and antegrade cerebral perfusion unless they have co-morbidities or low body weight calling for initial palliation with coarctation repair and pulmonary artery banding.

The role of primary catheter-based treatment of native coarctation is controversial because of the frequent need for reintervention and late aneurysm formation. Dijkema et al. report at least a 100% higher risk of reintervention and hypertension after non-surgical treatment of CoA [2]. Longer studies suggest up to a five-fold risk of aortic arch reintervention after percutaneous treatment of native CoA [4]. Accordingly, we think a catheter-based approach using a stent should only be reserved for older children (>15 years) and adults with localised CoA.
